# Supplementary material for: Microbiome histidine competition mediates dietary control of systemic imidazole propionate
Source: bioRxiv. 2026 Jun 23:2026.06.22.733842. Preprint. [Version 1] doi: 10.64898/2026.06.22.733842 (PMC13320830; doi:10.64898/2026.06.22.733842)
Supplement: Supplement 2 [file NIHPP2026.06.22.733842v1-supplement-2.pdf]

bioRxiv preprint doi: https://doi.org/10.64898/2026.06.22.733842; this version posted June 23, 2026. The copyright holder for this preprint (which was not certified by peer review) is the author/funder, who has granted bioRxiv a license to display the preprint in perpetuity. It is made available under a CC-BY 4.0 International license.

SUPPLEMENTAL MATERIAL

Microbiome histidine competition mediates dietary control of systemic imidazole propionate

Paola Nol Bernardino,[1,2] Christian Jacoby,[1,2] Isaac T. Younker,[1,2] Joshua Stemczynski,[1,2] Alexander S. Little,[1,2] Michael W. Mullowney,[1] Tess H. Brunner,[1,2] Joyce Ghali,[1,2] Matteo Fardin,[1] Kelsey Rose,[1] Ramanujam Ramaswamy,[1] Ashley M. Sidebottom,[1] Sarah A. Tersey,[3] Eric G. Pamer,[1,2] Raghavendra G. Mirmira,[3] Mark Mimee,[1,2,4] and Samuel H. Light[1,2]

[1]*Duchossois Family Institute, University of Chicago, Chicago, IL 60637, USA*
[2]*Department of Microbiology, University of Chicago, Chicago, IL 60637, USA*
[3]*Department of Medicine and the Diabetes Research and Training Center, University of Chicago, Chicago, IL 60637, USA*
[4]*Pritzker School of Molecular Engineering, University of Chicago, Chicago, IL 60637, USA*

Corresponding author/Lead contact: samlight@uchicago.edu

Supplemental Tables

Table S1. *O. symbiosa* normalized transcript counts when grown on histidine.

Table S2. Histidine pathway predictions for UHGG species representatives.

Table S3. Association between culture histidine depletion and oxidative histidine pathway reported by Han et al.

Table S4. Taxonomic composition and histidine pathway assignments for metagenomes in Figures S6 and S7.

Table S5. Differential gene expression in *O. symbiosa* grown on histidine with or without glutamate.

Table S6. Reference enzymes and homology thresholds for histidine pathway identification.

Table S7. Strains used in this study

bioRxiv preprint doi: https://doi.org/10.64898/2026.06.22.733842; this version posted June 23, 2026. The copyright holder for this preprint (which was not certified by peer review) is the author/funder, who has granted bioRxiv a license to display the preprint in perpetuity. It is made available under a CC-BY 4.0 International license.

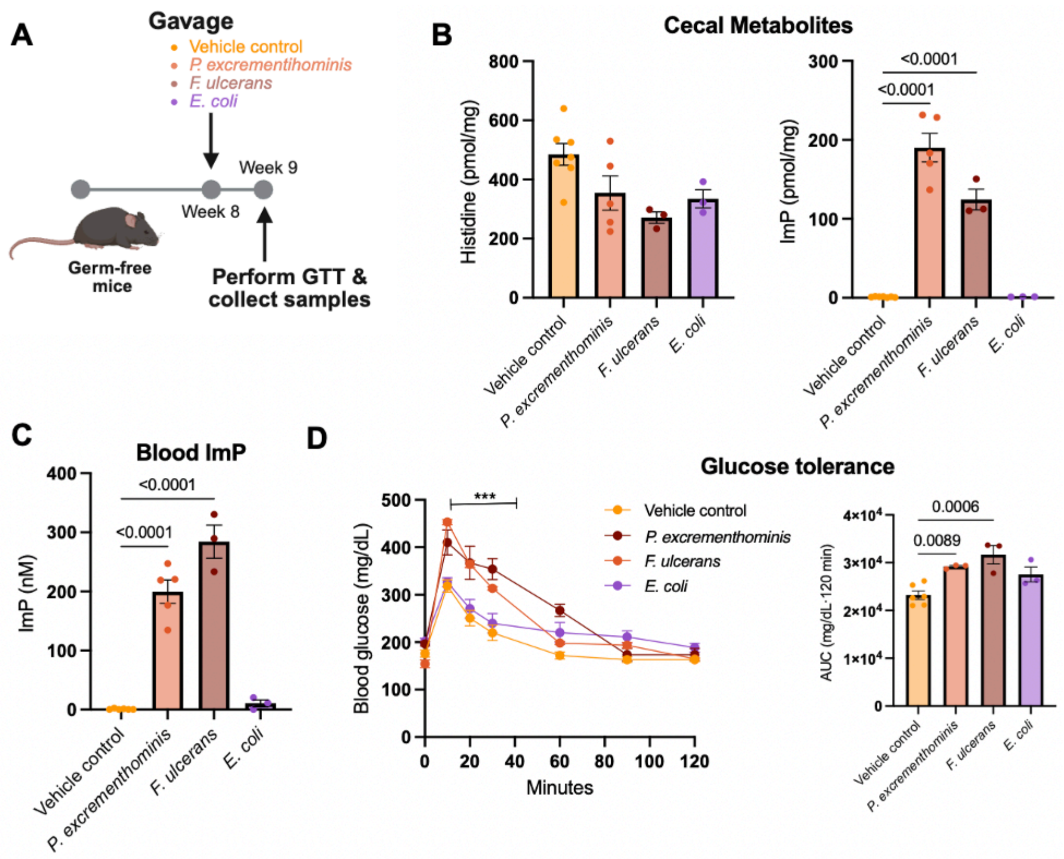

Figure S1. Monocolonization with reductive histidine pathway bacteria elevates systemic ImP and impairs glucose tolerance. (A) Experimental design for gnotobiotic monocolonization study. Mice were administered *F. ulcerans*, *P. excrementihominis*, *E. coli* (histidine pathway-lacking control), or vehicle (PBS). (B) Cecal metabolite concentrations determined by targeted LC-MS. (C) Blood ImP concentration. (D) Glucose tolerance assessed by intraperitoneal glucose tolerance test. All data shown as mean ± SEM and analyzed by one-way ANOVA with Tukey's post-hoc test.

bioRxiv preprint doi: https://doi.org/10.64898/2026.06.22.733842; this version posted June 23, 2026. The copyright holder for this preprint (which was not certified by peer review) is the author/funder, who has granted bioRxiv a license to display the preprint in perpetuity. It is made available under a CC-BY 4.0 International license.

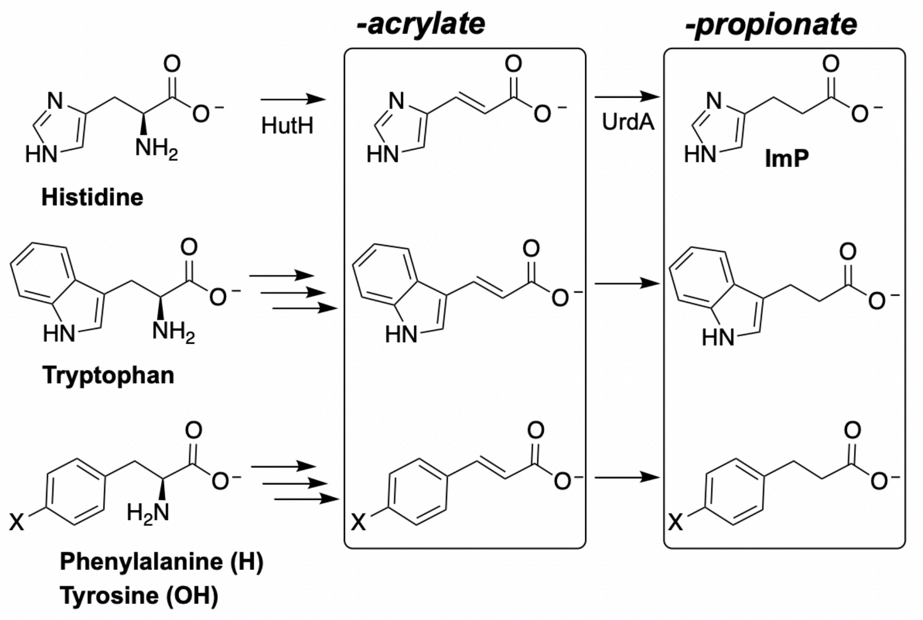

Figure S2. The reductive histidine pathway shares structural and mechanistic features with characterized reductive Stickland pathways. Comparison of histidine metabolism via HutH and UrdA with characterized phenylalanine, tyrosine, and tryptophan Stickland pathways. Conserved features include: (1) amino acid deamination to α,β-unsaturated acrylate intermediates, (2) reductase-mediated reduction to saturated products, and (3) generation of "-propionate" carboxylate products.

bioRxiv preprint doi: https://doi.org/10.64898/2026.06.22.733842; this version posted June 23, 2026. The copyright holder for this preprint (which was not certified by peer review) is the author/funder, who has granted bioRxiv a license to display the preprint in perpetuity. It is made available under a CC-BY 4.0 International license.

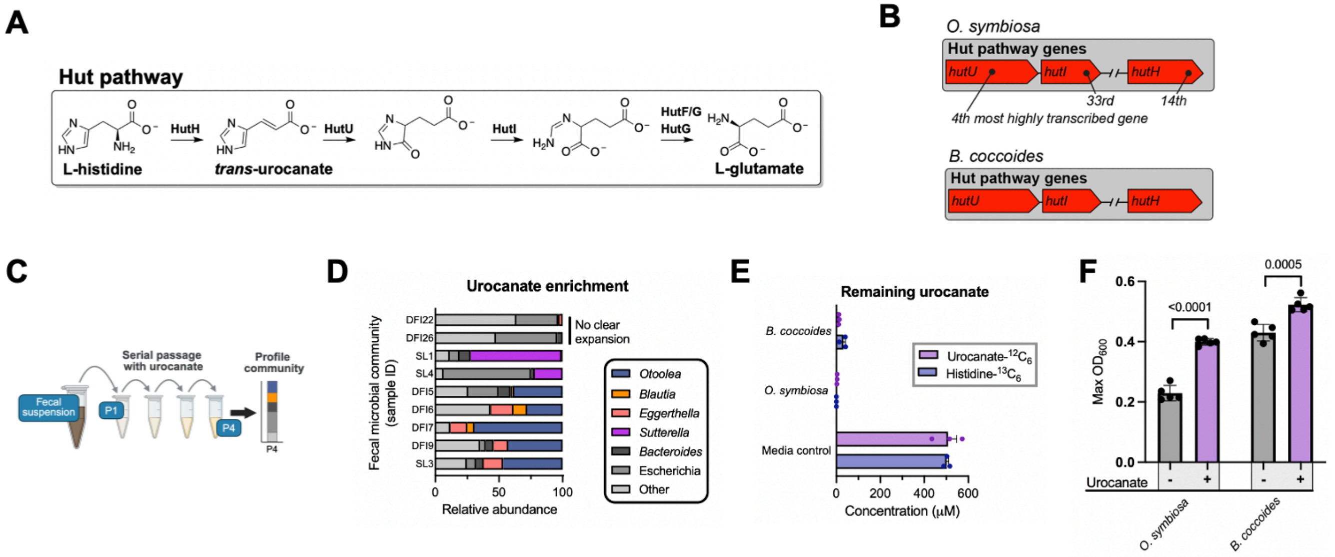

Figure S3. Evidence for Hut pathway-mediated histidine utilization in *O. symbiosa* and *B. coccoides*. (A) Enzymatic steps in histidine to glutamate conversion via the Hut pathway. The enzymes that catalyze the final step (HutG and/or HutF) in the pathway exhibit considerable species-level variability and consequently were not analyzed in our studies. (B) Genomic organization of Hut pathway genes in *O. symbiosa* and *B. coccoides*. Gene expression rankings based on normalized transcript abundance from 4,175 predicted *O. symbiosa* open reading frames from RNA-seq analysis of *O. symbiosa* cultured with histidine as the primary carbon source. (C) Experimental design for enriching urocanate-catabolizing bacteria from human fecal communities through serial passaging with urocanate as the primary substrate. (D) Community composition of human fecal microbial communities following urocanate enrichment passaging. (E) Remaining unlabeled urocanate and histidine-$^{13}C_6$ in culture media of *O. symbiosa* and *B. coccoides* supplemented with both substrates. (F) Growth of *O. symbiosa* and *B. coccoides* cultured with or without 10 mM urocanate supplementation. Data in E-F shown as mean ± SEM and analyzed by Two-tailed Welch's t-test.

bioRxiv preprint doi: https://doi.org/10.64898/2026.06.22.733842; this version posted June 23, 2026. The copyright holder for this preprint (which was not certified by peer review) is the author/funder, who has granted bioRxiv a license to display the preprint in perpetuity. It is made available under a CC-BY 4.0 International license.

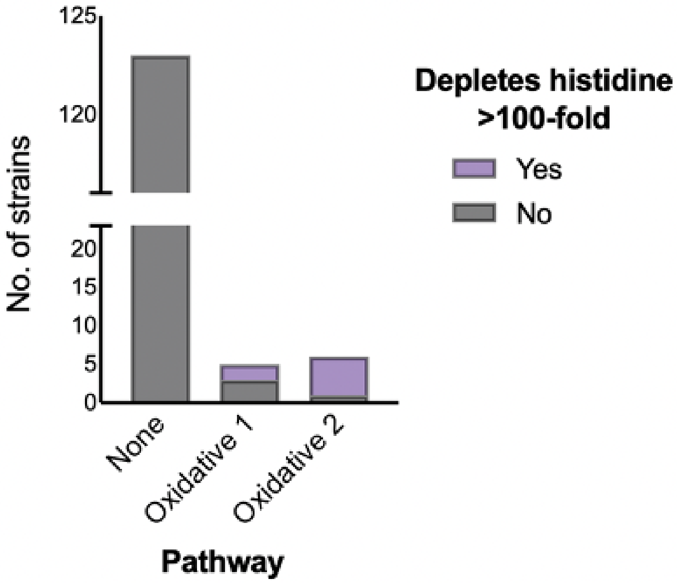

Figure S4. Histidine-depleting activity correlates with oxidative histidine pathway gene content in gut bacteria. Analysis of published metabolomics data (Han et al.) quantifying histidine depletion from culture media of individually grown gut bacterial strains. Strains are grouped by pathway genomic analysis of gene content: (1) none (no oxidative histidine pathway genes); (2) oxidative 1 (Hut pathway and methylaspartate pathway genes); (3) oxidative 2 (Hut pathway and 2-hydroxyglutarate pathway genes). Bars indicate the number of strains in each group that substantially depleted histidine (>100-fold reduction) from culture media. See **Table S3** for strain identities.

bioRxiv preprint doi: https://doi.org/10.64898/2026.06.22.733842; this version posted June 23, 2026. The copyright holder for this preprint (which was not certified by peer review) is the author/funder, who has granted bioRxiv a license to display the preprint in perpetuity. It is made available under a CC-BY 4.0 International license.

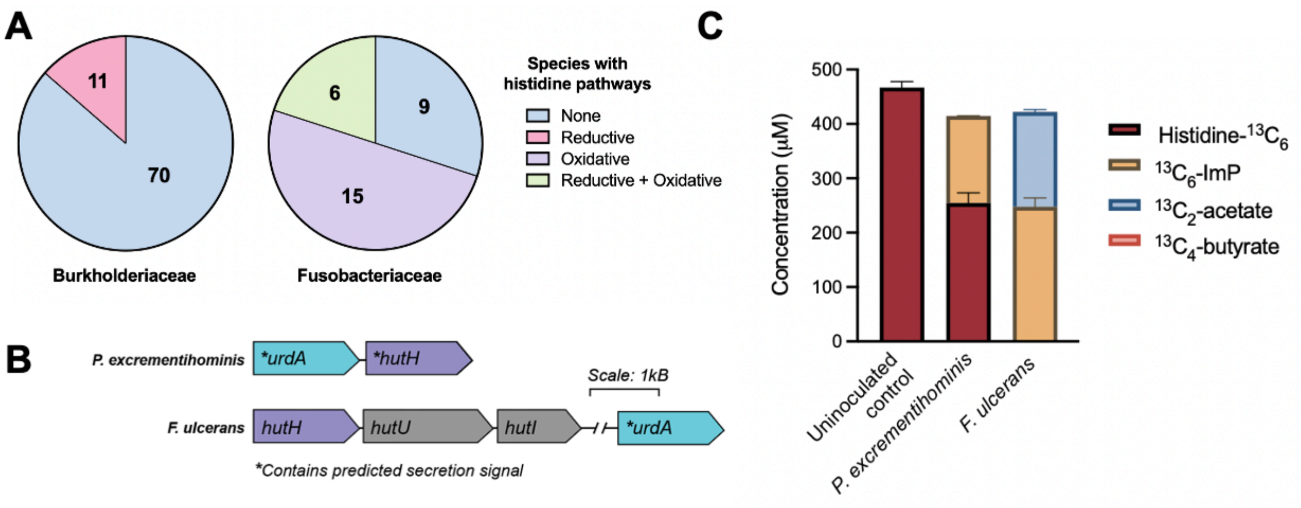

Figure S5. Distinct genomic organization of the reductive histidine pathway in reductive pathway-encoding bacteria. (A) Histidine pathway gene content in UHGG genomes from reductive pathway-encoding families Burkholderiaceae and Fusobacteriaceae. Candidate oxidative and reductive histidine pathways were identified using marker genes, with genomes categorized as: (1) None (no pathway genes), (2) Reductive (Reductive pathway only), (3) Oxidative (Oxidative pathway only), or (4) Reductive + Oxidative (both reductive and oxidative pathway). Genome counts per category are shown. See **Table S2** for strain identities. (B) Representative genetic loci showing reductive histidine pathway gene organization in Burkholderiaceae (*P. excrementihominis*) and Fusobacteriaceae (*F. ulcerans*). Burkholderiaceae lack the oxidative histidine pathway and *urdA* and *hutH* co-localize. In Fusobacteriaceae, *hutH* co-localizes with oxidative histidine pathway genes (*hutU* and *hutI*), while *urdA* occupies a separate genomic location. (C) ^{13}C-labeled metabolite production from *P. excrementihominis* and *F. ulcerans* cultures supplemented with histidine-$^{13}C_6$.

bioRxiv preprint doi: https://doi.org/10.64898/2026.06.22.733842; this version posted June 23, 2026. The copyright holder for this preprint (which was not certified by peer review) is the author/funder, who has granted bioRxiv a license to display the preprint in perpetuity. It is made available under a CC-BY 4.0 International license.

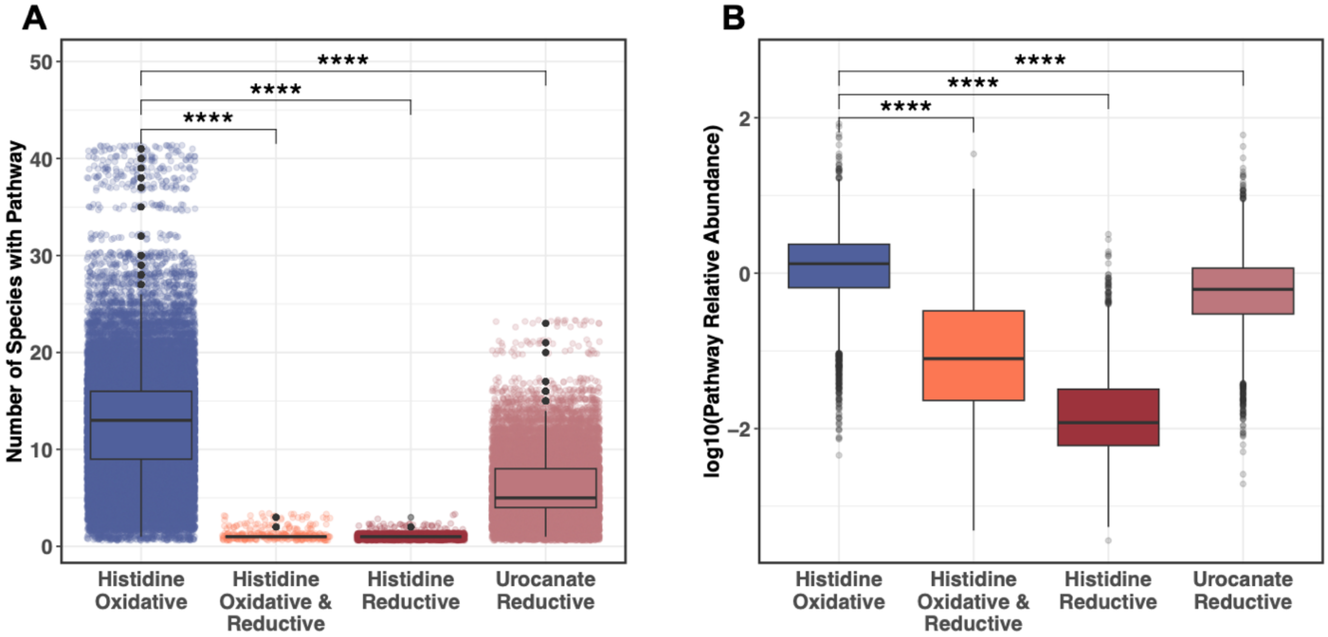

Figure S6. The oxidative histidine pathway is more prevalent and abundant than the reductive pathway in human gut microbiomes. Analysis of publicly available human fecal metagenomes showing distribution of bacterial histidine pathway types. Categories represent mutually exclusive pathway configurations as designated in **Table S2**: (1) Oxidative: species encoding Hut and glutamate fermentative pathways only; (2) Reductive: species encoding only reductive histidine pathway genes; (3) Oxidative & Reductive: species encoding genes for both pathways; (4) Urocanate Reductive: species encoding *urdA* without *hutH*, which potentially convert urocanate but not histidine to ImP. (A) Number of bacterial species in each pathway category per metagenome. Each point represents one metagenome. (B) Cumulative relative abundance of species in each pathway category per metagenome, calculated by summing the relative abundances of all species within each category. Statistical analysis by Wilcoxon rank-sum test with Benjamini-Hochberg correction for multiple testing.

bioRxiv preprint doi: https://doi.org/10.64898/2026.06.22.733842; this version posted June 23, 2026. The copyright holder for this preprint (which was not certified by peer review) is the author/funder, who has granted bioRxiv a license to display the preprint in perpetuity. It is made available under a CC-BY 4.0 International license.

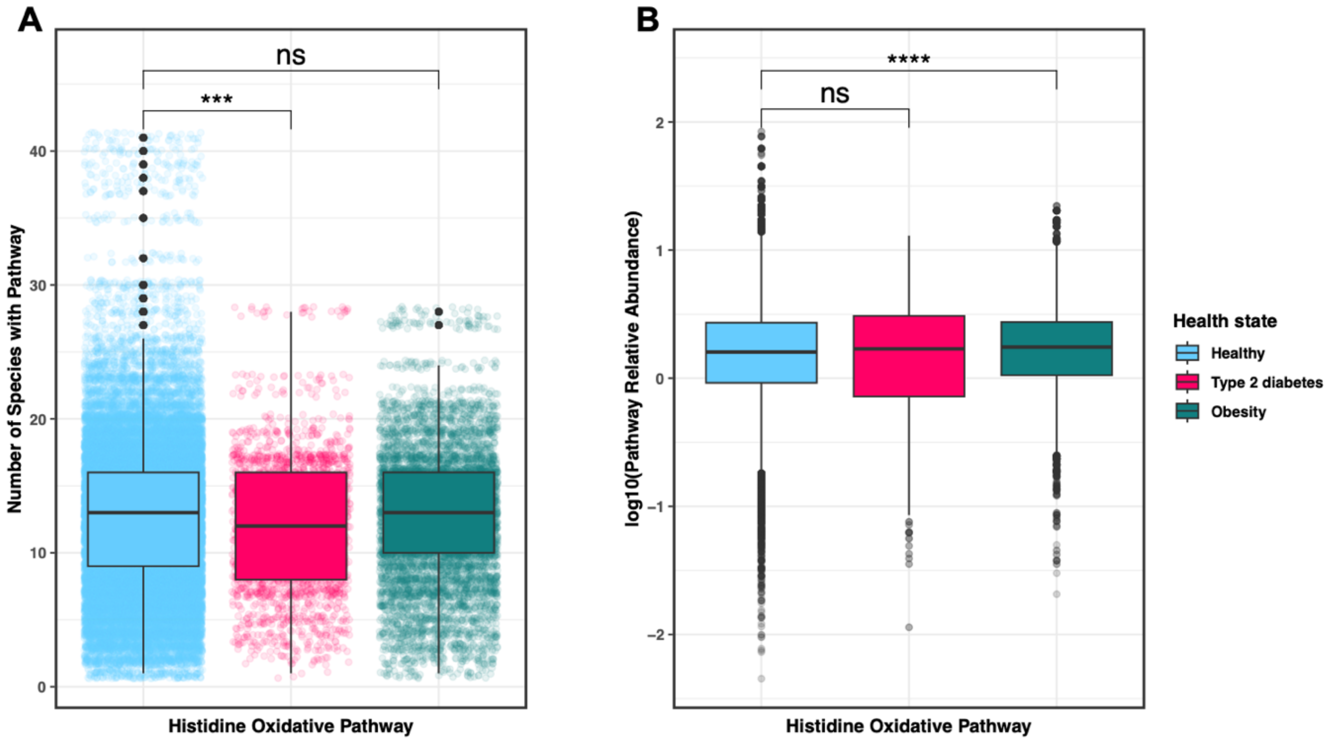

Figure S7. Oxidative histidine pathway bacteria show similar prevalence and abundance in healthy and cardiometabolic disease cohorts. (A) Analysis of fecal metagenomes from healthy controls, type 2 diabetes patients, and obese individuals, showing the number of bacterial species encoding oxidative histidine pathways detected per metagenome. Each point represents one individual. (B) Cumulative relative abundance of all oxidative pathway species per metagenome, calculated by summing the relative abundances of all species encoding the oxidative pathway within each sample. Statistical analysis by Wilcoxon rank-sum test with Benjamini-Hochberg correction for multiple testing.

bioRxiv preprint doi: https://doi.org/10.64898/2026.06.22.733842; this version posted June 23, 2026. The copyright holder for this preprint (which was not certified by peer review) is the author/funder, who has granted bioRxiv a license to display the preprint in perpetuity. It is made available under a CC-BY 4.0 International license.

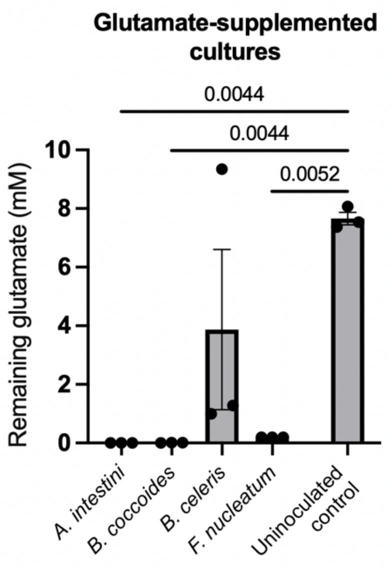

Figure S8. Oxidative pathway bacteria efficiently deplete glutamate while suppressing histidine consumption. Remaining glutamate in cultures of oxidative histidine pathway incubated with histidine-$^{13}C_6$ + glutamate, from conditions showing glutamate-mediated histidine consumption suppression shown in **Figure 3E**.

bioRxiv preprint doi: https://doi.org/10.64898/2026.06.22.733842; this version posted June 23, 2026. The copyright holder for this preprint (which was not certified by peer review) is the author/funder, who has granted bioRxiv a license to display the preprint in perpetuity. It is made available under a CC-BY 4.0 International license.

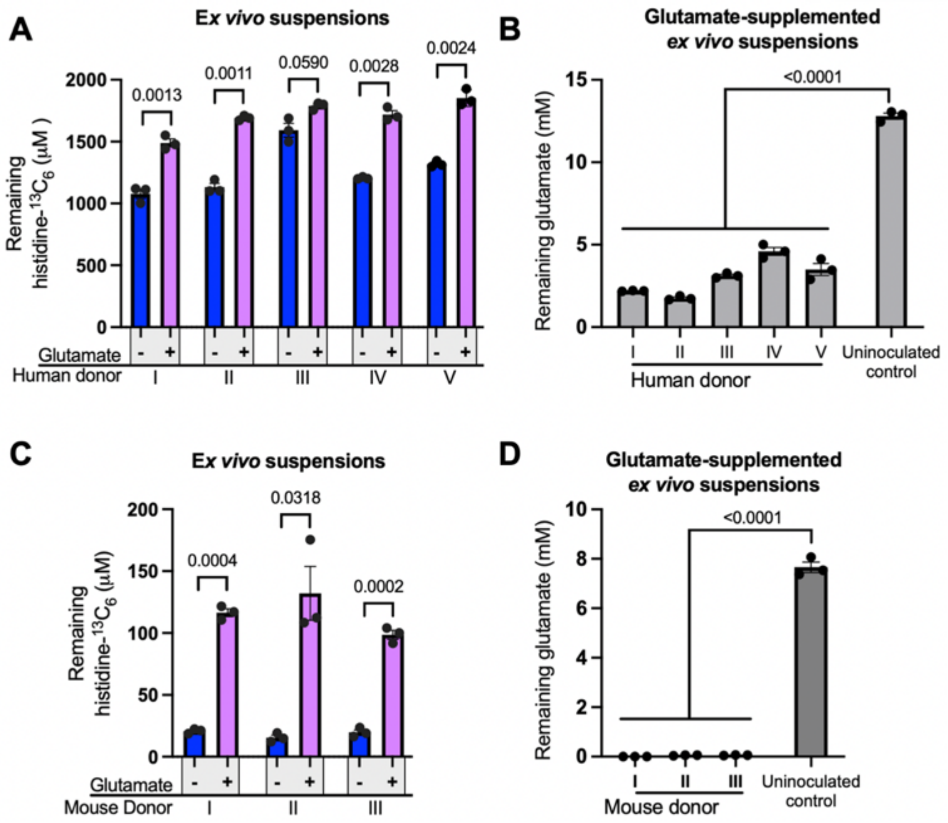

Figure S9. Glutamate consumption coincides with suppressed histidine depletion in natural microbial communities. Substrate consumption data from the ex vivo incubations shown in **Figure 4E** (human communities) and **Figure 4F** (mouse communities). (A) Remaining histidine-$^{13}C_6$ following *ex vivo* incubation of human fecal suspensions with histidine-$^{13}C_6$ ± glutamate. (B) Remaining glutamate following *ex vivo* incubation of human fecal suspensions with histidine-$^{13}C_6$ + glutamate. (C) Remaining histidine-$^{13}C_6$ following *ex vivo* incubation of mouse fecal suspensions with histidine-$^{13}C_6$ ± glutamate. (D) Remaining glutamate following *ex vivo* incubation of mouse fecal suspensions with histidine-$^{13}C_6$ + glutamate. All data shown as mean ± SEM and analyzed by Two-tailed Welch's t-test or one-way ANOVA with Tukey's post-hoc test.

bioRxiv preprint doi: https://doi.org/10.64898/2026.06.22.733842; this version posted June 23, 2026. The copyright holder for this preprint (which was not certified by peer review) is the author/funder, who has granted bioRxiv a license to display the preprint in perpetuity. It is made available under a CC-BY 4.0 International license.

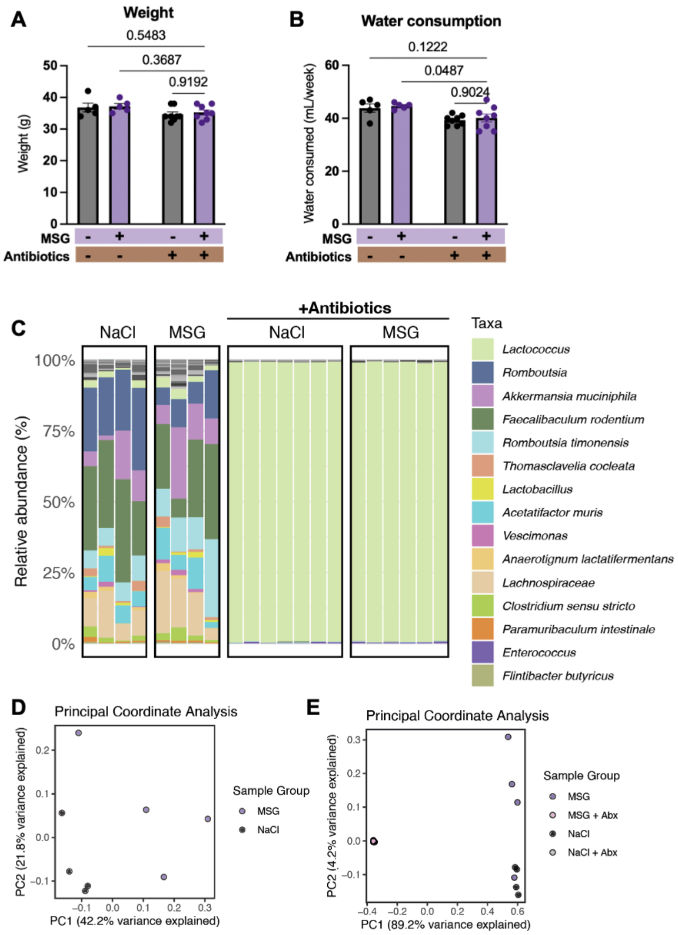

Figure S10. MSG supplementation has no major effects on body weight, water consumption, or microbiome composition. Physiological and microbiome data from the mouse MSG experiment shown in **Figure 5**. (A) Body weight and (B) weekly water consumption in mice receiving MSG or equimolar NaCl via drinking water with or without antibiotic treatment (Abx). Data in A-B shown as mean ± SEM and analyzed by one-way ANOVA. (C) Bacterial community composition determined by 16S rRNA amplicon sequencing. (D) Principal coordinate analysis of fecal 16S rRNA profiles based on Bray-Curtis dissimilarity of non-antibiotic-treated samples only, showing no significant separation between MSG and NaCl groups (Two-tailed Welch's t-test, $P > 0.05$). (E) Principal coordinate analysis (PCoA) of fecal 16S rRNA profiles based on Bray-Curtis dissimilarity, showing all treatment groups.
